# On Modern Progress in Ophthalmic Medicine and Surgery

**Published:** 1895-12-07

**Authors:** Robert Brudenell Carter

**Affiliations:** Consulting Ophthalmic Surgeon to St. George's Hospital.


### On Modern Progress in Ophthalmic Medicine and Surgery.

By Robert Brudenell Carter, F.R.C.S., Consulting Ophthalmic Surgeon to St. George's Hospital.

SQUINT (*Continued from page* 93).

When, on any of the grounds mentioned in the last paper, an operation for the cure of squint is decided upon, it is usually as desirable that it should not be unduly postponed as that it should not be performed too early. Operations on the adult, although they frequently result in obtaining what seems to a casual observer to be a good position, rarely so far overcome the long separation of the eyes as to lead to the restoration of perfect binocular vision, or to the power of easily maintaining combination of the eyes at any distance; and no results short of the attainment of both these objects can be regarded as fully accomplishing the desires of the surgeon. The best age is, I think, about eight years; and while I scarcely ever desire, except in the already-mentioned case of defective vision of the squinting eye, to perform the operation at an earlier period, I scarcely ever wish to defer it until a later one.

The operation for squint, as ordinarily performed, consists in the severance of the tendon of the muscle producing the deviation from its attachment to the sclera, so that it may retract, and gain a new attachment at some part of the eyeball posterior to the former one, from which its power to determine the position of the organ will be materially diminished.

It will be manifest that an operation upon a single muscle must sometimes produce only a very imperfect cure. Let us suppose that we are dealing with a case of common internal or convergent squint, apparently limited to the right eye, and three millimetres in extent. The effect of this condition will be, not that the centre of the pupil of each eye has a resting position at a millimetre and a half inside the central point of the lid opening, but that the centre of the left pupil is over this central point, while that of the right pupil stands three millimetres to the nasal side of it. If we divide the tendon of the right internal rectus to such an extent that it cannot produce as much convergence as before by as much as three millimetres, the eyes will be straight when the patient is looking towards a distant object directly in front of him, and the operation will be thought successful by a superficial observer. But, if these same eyes are turned towards a near object, as in reading, the power of the right eye to assume the position of convergence is found to be seriously limited, and it often squints divergently with reference to the near point looked at, not fixing it, and not conferring the benefits of binocular vision. At the same time, the globe having been allowed to come a little forward by the severance of one of the muscles which held it back, the eyelids will be somewhat more widely opened, and the eye more prominent than its fellow. For these reasons it was long the practice to divide the correction between the two eyes in all squints of sufficient magnitude, and a deviation of five millimetres was found by experience to admit, in most cases, of an operation on both eyes at once, that is to say, on both internal recti. In such cases parallelism was often retained after the double operation, and the power of divergence was not unduly diminished. In lesser squints, and especially of those of or under three millimetres, a double operation was very frequently followed by divergence, so that the last state of the patient was worse than the first. It became the accepted practice to operate on both eyes at once in large squints, but in small ones to operate on the affected eye only, and to wait three months or more before deciding whether more should be done. At the end of that time the position was sometimes found so nearly perfect that an operation on the other eye was avoided for fear it might lead to divergence; while, at the same time, the increased prominence of the formerly deviating eye was such as to diminish the cosmetic effects hoped for from the first operation. In girls with small squints the surgeon was often on the horns of a dilemma, in which an operation on one eye would hardly be sufficient, while an operation on both would certainly be excessive. The difficulty arose, in the main, from the want of any means by which the precise amount of retraction of the severed muscle could be controlled and determined.

The muscles that move the eyes, and for the present I confine myself to the four recti, take their rise from the bones of the skull at the apex of the orbit, and pass forward, embracing the eyeball in their course, but separated from it by a fibrous sheath or capsule, called the capsule of Tenon, which invests the eyeball. Nearly opposite the equator of the eye the four muscles perforate the capsule of Tenon to reach their points of insertion, but they carry with them a sheath derived from this capsule, which is continued over them almost to the eye itself, and to which they are united by a delicate web of connective tissue. If the attachment of the tendon to the sclera is severed close to the latter membrane, in front of the sheath derived from the capsule, the muscle is so held that its retraction is comparatively small in amount; while if the sheath itself be extensively divided, the retraction of the muscle is unrestrained, and may be very considerable. The ideal operation would be one in which the correction was divided between the two eyes in such a manner that the retraction of each of the divided muscles should be equal to half the total magnitude of the deviation.

For want of recognition of this principle, the consequences of many of the early operations for squint were as bad as, or even worse than, the deformities which they were intended to correct. The early operations were done by small curved knives, guided by directors, and the insertion of these was generally somewhat roughly managed, after free incisions of the conjunctiva and subconjunctival tissue, with the effect that the capsule of Tenon was freely divided both above and below the borders of its tendon, and that all the surrounding structures came more or less under the influence of the knife. As a rule, the severed muscle was left free to retract in a degree governed only by the contractility of its own fibres, and the surgeon was rather desirous to set the eye at liberty than to confine his proceedings within the limits which modern surgery has found it necessary to lay down.

